# Deaths and hospitalizations resulting from poisoning by prescription and over-the-counter drugs in Brazil

**DOI:** 10.11606/s1518-8787.2021055003551

**Published:** 2021-11-23

**Authors:** Fernanda Gross Duarte, Marcelo Neubauer de Paula, Nelzair Araújo Vianna, Maria Conceição Chagas de Almeida, Edson Duarte Moreira

**Affiliations:** I Fundação Oswaldo Cruz Instituto Gonçalo Moniz Salvador BA Brasil Pós-Graduação em Biotecnologia em Saúde e Medicina Investigativa. Instituto Gonçalo Moniz, Fundação Oswaldo Cruz. Salvador, BA, Brasil; II Grupo Hypera Pharma São Paulo SP Brasil Grupo Hypera Pharma. São Paulo, SP, Brasil; III Fundação Oswaldo Cruz Instituto Gonçalo Moniz Laboratório de Epidemiologia Molecular e Bioestatística Salvador BA Brasil Fundação Oswaldo Cruz. Instituto Gonçalo Moniz. Laboratório de Epidemiologia Molecular e Bioestatística. Salvador, BA, Brasil

**Keywords:** Medicamentos sem Prescrição, envenenamento, Medicamentos sob Prescrição, envenenamento, Hospitalização, Envenenamento, mortalidade

## Abstract

**OBJECTIVE:**

To estimate the incidence of hospitalizations considering drug intoxication and the mortality of these diseases in Brazil, given trends from 2009 to 2018.

**METHODS:**

Data on hospital admissions and deaths come from DATASUS and demographic data from the Brazilian Institute of Geography and Statistics (IBGE). Hospital admissions with *Autorização para Internação Hospitalar* (AIH - Authorization for Hospital Admission) indicated as a procedure “treatment of intoxication or poisoning due to exposure to drugs and substances for non-drug use” were selected, with only cases of hospitalization due to drug intoxication being analyzed. The incidence of hospitalization and mortality were calculated separately for intoxications caused by *medicamentos com prescrição* (MRx - prescription drugs) and *medicamentos isentos de prescrição* (MIP - over-the-counter drugs). Rates were further stratified by sex, age group, and region of residence in Brazil. Trend analysis was performed by generalized linear regression using the Prais-Winsten method.

**RESULTS:**

MRx caused most hospitalizations (97%), with mortality approximately 50 times higher when compared to hospitalizations for MIP. The incidence trend in hospitalizations for MRx was stationary and mortality increased during the study period, whereas the trend in mortality and in the incidence of hospitalizations for MIP decreased.

**CONCLUSIONS:**

Hospitalizations for drug intoxication, especially those caused by MRx, have a great impact and importance on public health considering that prevention is possible.

## INTRODUCTION

Drug intoxication results from exposure to a drug at doses higher than those usually used for prophylaxis, diagnosis, treatment, or for modification of physiological functions. This may result in different signs and symptoms, depending on the type of drug and drug metabolism^1^. Intoxication can occur unintentionally or accidentally, when it results from self-medication, dosage error, inadequate therapy, medication change, or involuntary ingestion; or intentional, when related to abuse, misuse, or self-injury^2^. Severe cases of poisoning, leading to hospital admissions and even death, constitute an important public health problem^3^.

Between 2009 and 2018, the *Sistema Nacional de Informações Tóxico-Farmacológicas* (Sinitox - National Toxic-Pharmacological Information System) registered 254,135 cases of poisoning in Brazil, with a total of 710 deaths (0.28%). Medicines were the main cause of intoxication among all the agents notified, or about 29% of cases in the period, followed by venomous animals, household cleaning products, and pesticides^4^. Since 1994, pharmaceuticals occupy the first place in the ranking of intoxications and the second place in the number of deaths. In 2016, according to data from Sinitox, accidental poisoning was one of the main causes of occurrences with medications, representing about a third of reported cases (32.7%)^4^.

In most developed countries, the situation is similar to that in Brazil, with medicines leading the causes of exogenous intoxication^5^. In the United States of America (USA), national data from 2018 indicate that more than two million cases of intoxication were caused by medicines^6^. Additionally, there was an increasing trend in mortality rates from drug poisoning between 1994 and 2010, in all regions and demographic groups in the USA, with accidental poisoning being the main cause (71%)^7^.

In Brazil, drugs can be classified as *Medicamentos com prescrição* (MRx - prescription drugs) or *Medicamentos sentos de prescrição* (MIP - over-the-counter drugs). This classification was established for the first time in Brazilian sanitary legislation, in 1973, when the sanitary control of the medicine trade was regulated. In this case, the concept of over-the-counter drugs relates to not depend on prescription drugs, establishing differences regarding the labeling and advertising of prescribed products^8^. In 2016, the *Agência Nacional de Vigilância Sanitária* (Anvisa - National Health Surveillance Agency) more clearly defined the criteria for a drug to be classified as “prescription-free”, including 33 therapeutic groups, with exceptions^9^. The criteria for the inclusion of drugs in the MIP list take into account the following aspects: marketing time, drug safety, identifiable symptoms, use for a short period of time, being manageable by the patient, presenting low-risk potential, and not presenting dependence^9^.

We intend to estimate the incidence of hospitalizations for drug intoxication, generally and according to the type of drug (prescription or exempt), as well as the mortality of these diseases in Brazil, describing the trends observed in these estimates in the period between 2009 and 2018.

## METHODS

This is a retrospective study to determine the rates of hospital admissions and deaths caused by drug intoxication in Brazil. A review of data on drug poisoning was carried out in the *Base do Sistema Único de Saúde* (Datasus - Unified Health System Database) during 10 years (date of exit: 2009 to 2018), including information such as gender, age, place of residence, and type of drug intoxication. Results for intoxications caused by prescription drugs are presented separately from those caused by over-the-counter drugs.

Data on admissions and deaths are from the DATASUS portal and were extracted by the *TabWin software*, provided by DATASUS. Demographic data were obtained from the 2010 Census and from inter-census estimates for other years, made available through the data portal of the Brazilian Institute of Geography and Statistics (IBGE)^10^.

Initially, hospital admissions reported between 2009 and 2018, which the *Autorização para Internação Hospitalar* (AIH - Authorization for Hospital Admission) requested as a procedure, were selected for “Treatment of intoxication or poisoning due to exposure to medication and substances for non-drug use”. Subsequently, only confirmed admissions in this procedure were kept; admissions considered for another purpose in the discharge/death report were excluded. Cases of drug intoxication were classified according to therapeutic groups and set apart by prescription or over-the-counter, using information from the primary and secondary International Classification of Diseases (ICD). We analyzed cases resulting from drug intoxication in this article.

We reckoned the incidence of hospitalization by dividing the number of hospitalizations that occurred in the study population by the number of inhabitants corresponding to the period and place analyzed. Similarly, mortality rates were calculated by dividing the number of deaths by the total population in each study period. Since the crude rates can be influenced by the age structure of populations from different regions and at different time periods, the estimated rates were standardized by age group and direct method, using a the standard population suggested by the World Health Organization (WHO 2000–2025), thus allowing the analysis of trends and comparisons of these data^11^. The analysis of frequencies, rates of hospitalizations, and deaths was performed according to the type of drug intoxication classified as caused by prescription drugs (MRx) or over-the-counter drugs (MIP). We stratified the rates by sex, age group, and region of residence in Brazil. Generalized linear regression performed the trend analysis of the time series by using the Prais-Winsten method^12^, with correction for the first-order autocorrelation effect^13^. We considered the stationary trend of hospital admissions/deaths when p > 0.05; declining when p < 0.05 and negative regression coefficient; or ascending when p < 0.05 and positive regression coefficient^14^. Statistical analyzes were performed using the Stata statistical program (Stata Statistical Software: Release 16. College Station, TX: StataCorp LLC).

## RESULTS

Between 2009 and 2018, Brazil had 85,811 hospital admissions due to drug poisoning; MRx poisoning caused 97% of them and MIP caused 3%. The mean incidence of hospitalizations for MRx (4.16 per 100 thousand inhabitants) was much higher than that for MIP (0.13 per 100 thousand inhabitants), RR = 32.8 (95%CI 28.9–37.1). Hospitalizations accounted for 2,644 deaths (3.08%) during the study period. The mortality rate was higher for MRx poisoning (3.11%) than for MIP poisoning (1.93%).

Table 1 shows the frequency of hospital admissions and deaths caused by drug intoxication according to the region and other sociodemographic characteristics. The majority of hospitalizations occurred in females both in MRx poisoning (56.7%) and in those by MIP (54.9%). MRx hospitalizations had a higher mean incidence in females (4.61 per 100,000) compared to males (3.67 per 100,000), but this difference was lower in hospitalizations for MIP. In contrast, females accounted for only a quarter of deaths in admissions for MIP and just under half of those in admissions for MRx. White people were most commonly reported in admissions for MRx and MIP and in almost a third of cases had no information of color reported. The Southeast region had the highest number of hospitalizations for MRx, followed by the South and Midwest regions. The regional distribution of admissions for MIP was similar (Table 1).

**Table 1:** Frequency of hospitalizations and deaths by type of drug intoxication, Brazil, 2009–2018.

		Drugs with Prescription (MRx)				Over-the-counter drugs (MIP)				Total			
		Hospitalizations		Deaths		Hospitalizations		Deaths		Hospitalizations		Deaths	
		n	(%)	n	%	n	%	n	%	n	%	n	%
Total		83,275	100	2,595	100	2,536	100	49	100	85,811	100	2,644	100
Sex													
	Female	47,252	56.7	1,267	48.8	1,391	54.9	13	26.5	48,643	56.7	1,280	48,4
	Male	36,023	43.3	1,328	51.2	1,145	45.1	36	73.5	37,168	43.3	1,364	51.6
Age group													
	< 5	7,600	9.1	40	1.5	529	20.9	4	8.2	8,129	9.5	44	1.7
	5–9	3,399	4.1	14	0.5	194	7.6	1	2.0	3,593	4.2	15	0.6
	10–14	4,623	5.6	29	1.1	144	5.7	0	0	4,767	5.6	29	1.1
	15–19	8,468	10.2	129	5.0	279	11.0	2	4.1	8,747	10.2	131	5.0
	20–29	15,679	18.8	401	15.5	476	18.8	7	14.3	16,155	18.8	408	15.4
	30–39	14,851	17.8	504	19.4	304	12.0	10	20.4	15,155	17.7	514	19.4
	40–49	11,857	14.2	543	20.9	257	10.1	4	8.2	12,114	14.1	547	20.7
	50–59	7,497	9.0	401	15.5	155	6.1	7	14.3	7,652	8.9	408	15.4
	60–69	4,331	5.2	244	9.4	88	3.5	6	12.2	4,419	5.1	250	9.5
	70 or more	4,970	6.0	290	11.2	110	4.3	8	16.3	5,080	5.9	298	11.3
Skin color/race													
	White	32,814	39.4	921	36	911	35.9	11	22.4	33,725	39.3	932	35.2
	Brown	22,076	26.5	704	27	768	30.3	14	28.6	22,844	26.6	718	27.2
	Black	2,787	3.3	93	4	77	3.0	3	6.1	2,864	3.3	96	3.6
	Other	835	1.1	25	1	24	1.0	0	0	859	1.1	25	1.0
	Not informed	24,763	29.7	852	33	756	29.8	21	42.9	25,519	29.7	873	33.0
Regions													
	North	3,868	4.6	108	4.2	142	5.6	5	10.2	4,010	4.7	113	4.3
	Northeast	7,064	8.5	172	6.6	317	12.5	5	10.2	7,381	8.6	177	6.7
	Midwest	14,375	17.3	621	23.9	545	21.5	20	40.8	14,920	17.4	641	24.2
	Southeast	41,522	49.9	1,278	49.2	977	38.5	11	22.4	42,499	49.5	1,289	48.8
	South	16,446	19.7	416	16.0	555	21.9	8	16.3	17,001	19.8	424	16.0
Character of admission													
	Urgency	81,513	97.9	2,536	97.7	2,474	97.6	49	100	83,987	97.9	2,585	97.8
	Elective	1,762	2.1	59	2.3	62	2.4	0	0	1,824	2.1	59	2.2

Incidence of hospitalizations for MRx: 4.61 per 100,000 (in women) and 3.67 per 100,000 (in men).

Incidence of hospitalizations for MIP: 0.14 per 100,000 (in women) and 0.12 per 100,000 (in men).

Mortality from MRx: 123 per million (in women) and 135 per million (in men).

Mortality from MIP: 1.3 per million (in women) and 3.7 per million (in men).

The incidence of hospitalization and mortality by age group are shown in Table 2. Poisonings by MRx and MIP had a higher incidence of hospitalizations in children under 5 years of age, decreasing in frequency with increasing age, especially in hospitalizations for MIP. Mean mortality in the period studied was higher in hospitalizations for MRx than for MIP, RR = 52.0 (95%CI 21.5–126.0). 60 years or older individuals had the highest mortality rate in MRx and MIP poisonings, followed by the under-5 group in admissions for MIP.

**Table 2:** Incidence of hospitalization and mortality from drug intoxication by age group, Brazil, 2009–2018.

		Year										Annual average
		2009	2010	2011	2012	2013	2014	2015	2016	2017	2018	
**Hospitalization** a												
MRx (age group)												
	0 to 4 years	7.62	6.10	5.37	5.99	5.57	5.67	4.88	5.07	5.27	5.67	**5.72**
	5 to 19	4.47	3.23	3.32	3.25	3.25	2.95	2.86	2.83	3.85	4.40	**3.44**
	20 to 59 years	7.00	4.58	4.40	4.49	4.32	3.90	3.66	3.62	3.99	4.45	**4.44**
	60 years or older	4.49	3.55	3.37	3.09	3.23	3.45	3.44	3.26	3.66	3.51	**3.50**
	**Total**	**6.10**	**4.22**	**4.07**	**4.11**	**4.00**	**3.73**	**3.52**	**3.48**	**3.99**	**4.37**	**4.16**
MIP (age group)												
	0 to 4 years	0.58	0.27	0.42	0.42	0.49	0.41	0.34	0.32	0.43	0.30	**0.40**
	5 to 19	0.15	0.11	0.08	0.13	0.13	0.14	0.15	0.13	0.13	0.14	**0.13**
	20 to 59 years	0.20	0.11	0.12	0.10	0.11	0.09	0.09	0.08	0.08	0.08	**0.11**
	60 years o older	0.14	0.08	0.10	0.10	0.05	0.08	0.08	0.06	0.04	0.04	**0.08**
	**Total**	**0.21**	**0.12**	**0.13**	**0.13**	**0.13**	**0.12**	**0.12**	**0.11**	**0.10**	**0.10**	**0.13**
**Mortality** b												
MRx (age group)												
	0 to 4 years	66	37	22	15	7	23	15	23	38	53	**30**
	5 to 19	38	26	30	33	35	31	34	32	57	44	**36**
	20 to 59 years	221	137	155	155	148	152	155	160	164	191	**164**
	60 years or older	214	151	134	181	207	215	215	173	239	247	**198**
	**Total**	**162**	**103**	**112**	**119**	**119**	**124**	**126**	**125**	**143**	**159**	**129**
MIP (age group)												
	0 to 4 years	7.4	14.8	0	0	0	7.7	0	0	0	0	**3.0**
	5 to 19	0	0	0	0	6.2	0	0	0	0	0	**0.6**
	20 to 59 years	5.6	3.7	5.4	1.8	0.9	2.7	0.9	0	3.4	0.8	**2.5**
	60 years or older	4.5	8.9	13.0	3.9	0	7.3	0	10.2	3.3	3.1	**5.4**
	**Total**	**4.1**	**4.1**	**4.6**	**1.5**	**2.0**	**3.0**	**0.5**	**1.5**	**2.4**	**1.0**	**2.5**

MIP: over-the-counter medications; MRx: prescription drugs.

a Incidence of hospitalization per 100,000 inhabitants.

b Mortality per 100 million inhabitants.

Table 3 shows the incidence of hospitalization and mortality, standardized for the distribution of the world population (WHO 2000–2025), according to the regions of Brazil. During the study period, the highest mean incidence of hospitalizations for MRx occurred in the south and southeast regions, while the north and northeast regions had the lowest rates. In hospitalizations for MIP, the mean incidence was higher in the midwest and south regions. Similarly, the north and northeast regions had the lowest rates. We observed higher mortality for MRx poisonings in the southeast and south regions, whereas MIP had a higher rate in the northeast region.

**Table 3:** Incidence of hospitalization and mortality from drug intoxication by region, Brazil, 2009–2018.

		Year										Annual average	
		2009	2010	2011	2012	2013	2014	2015	2016	2017	2018		
**Hospitalization** a												
MRx (by region)												
	North	3.23	2.54	2.80	3.72	3.71	1.62	1.29	1.17	1.52	1.33	**2.29**
	Central-West Region	9.28	5.91	5.34	5.17	4.81	3.84	2.99	3.49	3.94	3.63	**4.84**
	Northeast	4.83	2.83	2.73	2.79	2.42	2.34	2.18	2.00	2.09	2.43	**2.66**
	Southeast	6.64	5.09	5.08	4.88	4.85	4.94	4.38	4.61	5.18	5.76	**5.14**
	South	8.35	5.53	4.81	4.94	5.35	5.38	4.96	5.81	7.18	7.72	**6.00**
	**Brazil**	**6.10**	**4.23**	**4.08**	**4.10**	**3.99**	**3.72**	**3.49**	**3.47**	**3.98**	**4.36**	**4.15**
MIP (by region)												
	North	0.13	0.05	0.06	0.10	0.11	0.12	0.09	0.02	0.08	0.04	**0.08**
	Central-West Region	0.57	0.26	0.21	0.20	0.19	0.24	0.10	0.11	0.13	0.15	**0.22**
	Northeast	0.17	0.11	0.15	0.10	0.11	0.08	0.09	0.07	0.05	0.06	**0.10**
	Southeast	0.17	0.09	0.10	0.12	0.12	0.12	0.12	0.12	0.10	0.09	**0.12**
	South	0.27	0.17	0.15	0.21	0.17	0.18	0.21	0.19	0.20	0.21	**0.20**
	**Brazil**	**0.21**	**0.12**	**0.13**	**0.13**	**0.14**	**0.13**	**0.12**	**0.11**	**0.11**	**0.11**	**0.13**
**Mortality** b												
MRx (by region)												
	North	68	80	58	74	67	49	53	48	128	53	**68**
	Central-West Region	222	130	111	113	177	103	116	71	148	111	**130**
	Northeast	224	76	105	131	120	103	91	135	101	147	**123**
	Southeast	153	137	149	154	155	191	148	176	194	226	**168**
	South	205	136	123	124	132	157	134	139	236	249	**164**
	**Brazil**	**158**	**101**	**108**	**115**	**115**	**120**	**121**	**120**	**139**	**155**	**125**
MIP (by region)												
	North	0	5.73	0	0	10.94	5.41	0	0	0	5.52	**2.76**
	Central-West Region	7.07	0	25.42	0	0	6.60	0	0	0	0	**3.91**
	Northeast	5.60	13.52	6.79	1.83	1.54	6.11	0	3.02	3.56	2.99	**4.50**
	Southeast	5.70	1.22	2.05	2.03	0	1.18	1.08	1.96	1.15	0	**1.64**
	South	0	0	7.14	3.54	2.98	3.48	0	5.78	9.14	0	**3.21**
	**Brazil**	**4.09**	**4.24**	**4.43**	**1.46**	**2.06**	**3.02**	**0.46**	**1.49**	**2.32**	**0.94**	**2.45**

MIP: over-the-counter medications; MRx: prescription drugs.

a Incidence of hospitalization per 100,000 inhabitants standardized for world population distribution (WHO, 2000–2025).

b Mortality per 100 million population standardized for world population distribution (WHO, 2000–2025).

The total number of hospitalizations and deaths, the regression coefficients, and the trend of hospitalizations and mortality by type of drug intoxication by region are presented in Table 4. In the period from 2009 to 2018, the trend in the incidence of hospitalizations due to MRx drug poisoning was stationary, ascending in the Southern region and decreasing in the Midwest and Northeast regions. While the incidence of hospitalizations for MIP had a decreasing trend in Brazil, especially in the Midwest and Northeast regions. The MRx poisoning mortality tended to ascended in Brazil, including in the South and Southeast regions. The mortality of poisoning by MIP, on the other hand, showed a decreasing trend in the country.

**Table 4:** Hospitalizations and deaths, regression coefficient and trend of hospitalizations and mortality from drug intoxication by region, Brazil, 2009–2018.

		N	Coefficient	95%CI	p	Estimated date
**Hospitalization**						
MRx (by region)						
	North	3,868	−0.268	−0.586; 0.050	0.087	Stationary
	Central-West Region	7,064	−0.224	−0.407; −0.041	0.023	**Decreasing**
	Northeast	14,375	−0.080	−0.146; −0.014	0.024	**Decreasing**
	Southeast	41,522	0.124	−0.020; 0.268	0.082	Stationary
	South	16,446	0.514	0.328; 0.700	0.000	**Upwardly**
	Brazil	83,275	0.049	−0.072; 0.169	0.371	Stationary
MIP (by region)						
	North	142	−0.002	−0.0153; 0.010	0.605	Stationary
	Central-West Region	317	−0.014	−0.026; −0.002	0.025	**Decreasing**
	Northeast	545	−0.010	−0.011; −0.009	0.000	**Decreasing**
	Southeast	977	−0.000	−0.003; 0.002	0.705	Stationary
	South	555	0.004	−0.000; 0.009	0.052	Stationary
	Brazil	2,536	−0.004	−0.005; −0.002	0.001	**Decreasing**
**Fatalities**						
MRx (by region)						
	North	108	0.283	−5.478; 6.045	0.911	Stationary
	Central-West Region	172	−3.446	−9.001; 2.109	0.186	Stationary
	Northeast	621	1.772	−1.879; 5.423	0.289	Stationary
	Southeast	1,278	6.680	3.959; 9.401	0.001	**Upwardly**
	South	416	15.527	6.757; 24.297	0.004	**Upwardly**
	Brazil	2,595	5.885	3.635; 8.136	0.000	**Upwardly**
MIP (by region)						
	North	108	0.283	−5.478; 6.045	0.911	Stationary
	Central-West Region	172	−3.446	−9.001; 2.109	0.186	Stationary
	Northeast	621	1.772	−1.879; 5.423	0.289	Stationary
	Southeast	1,278	6.680	3.959; 9.401	0.001	**Upwardly**
	South	416	15.527	6.757; 24.297	0.004	**Upwardly**
	Brazil	2,595	5.885	3.635; 8.136	0.000	**Upwardly**

MIP: over-the-counter medications; MRx: prescription drugs.

## DISCUSSION

In the decade evaluated in this study (2009–2018), MRx caused most hospitalizations (97%) for drug intoxication, an incidence 32 times greater than that of hospitalizations for MIP. Despite the large difference indicating the preponderant role of MRx compared to MIP in severe cases, commerce data during the second half of the studied decade (2014–2018) showed that fewer doses of MRx (7.2 billion) were sold than of MIP (7.7 billion)^15^. Prescription exceptions are mainly based on the low toxicity potential of these products, which have reversible adverse reactions after discontinuation of use and serious reactions only with the administration of large amounts of the product, outside the safe therapeutic window^16^. This safety profile is possibly responsible for the lower number of serious poisonings and hospitalizations caused by MIP. The much higher mortality observed in hospitalizations for MRx, about 50 times higher, supports this argument.

ANVISA is responsible for regulating advertising, publicity, information, and other practices with the purpose of disclosing or commercially promoting medicines^17^. The agency includes materials prepared by the pharmaceutical industry for prescribers and dispensers. The results of this research, indicating much higher mortality in the use of prescription drugs, have important implications that justify the improvement of this regulation to prevent self-medication and the use of drugs in a non-rational way.

Hospitalizations were more common in females, while mortality was higher in males, both for MIP and MRx poisoning. Mota et al. (2012) analyzed the 1996 to 2005 period and also observed a higher prevalence of deaths from drug poisoning in men^18^. Children had more hospitalizations for drug intoxication in under 5 years of age, regardless of the type of medication. Accidental drug ingestion is more common in this age group^19,20^. Moreover, the lower body weight in this group makes them more vulnerable to intoxication with relatively lower doses^21,22^. Mortality rates were higher in the older adults, similar to what was observed in other studies^23,24^. It is possible that the more frequent use of drugs and the increased susceptibility to toxicity, associated with the decrease in the ability to metabolize and excrete drugs due to advancing age, may have contributed to the higher mortality observed in older adults^24,25^.

The incidence of hospitalizations for MIP and MRx varied in Brazil, wherein the South, Midwest, and Southeast regions had the highest rates and the North and Northeast regions the lowest. Mortality from poisonings varied according to the region of Brazil. In the southeast and south there were the highest mortality rates for MRx, while the Northeast and Midwest regions had the highest mortality for MIP. There is evidence in Brazil of the association between greater purchasing power and greater use of medicines^26^. This may explain the higher and growing rates of drug intoxication in the south and southeast regions, where the per capita income of the population is higher than in other regions of the country^27^. Thus, the differences observed may be consequences of inequalities in the availability and access to drugs. They can also be consequences of differences in the coverage of health services responsible for reporting cases and deaths from drug poisoning in different regions in Brazil.

The trend in the incidence of hospitalizations for MRx was stationary, but mortality increased during the decade, whereas we observed a decreasing trend in mortality and in the incidence of hospitalizations for MIP in the same period. The epidemiological profile of the Brazilian population has been changing with an increase in the prevalence of chronic diseases related to the more frequent use of medications^28,29^. Polypharmacy has become a problem among older adults. In Brazil, it is estimated that 82% of people over 60 years old use at least one medication^30^. Although these drugs can increase quality and life expectancy, they can also pose risks to this population due to drug interactions and the change in physiology caused by senescence^31^. This may be one of the factors responsible for maintaining the incidence of hospitalizations and the increase in mortality in cases of MRx poisoning.

### Limitations and Merits

The retrospective nature of this study, based on a pre-existing database whose available information is limited, hampered the investigation of certain characteristics related to the circumstances, causes, and treatment of hospitalizations for drug intoxication. Moreover, the information refers only to public health services, not covering private services. Therefore, the rates produced are underestimated. However, national coverage of the data, including a period of several years, allowed for comparisons between different regions and provided trend measures for the incidence of hospitalizations for drug intoxication, as well as for mortality.

## CONCLUSIONS

Hospitalizations for drug intoxication have a great impact and importance on public health, deserving attention especially for its potentially preventable nature. Despite greater access to prescription-free medicines, almost all hospitalizations and deaths in Brazil between 2009 and 2018 occurred as a result of intoxication caused by prescription-only medicines. Our results reinforce the need for analytical studies to identify the determinants and prevent the occurrence of drug intoxications.
