# Supplementary material for: Deaths and hospitalizations resulting from poisoning by prescription and over-the-counter drugs in Brazil
Source: Rev Saude Publica. 2021 Nov 23;55:81. doi: 10.11606/s1518-8787.2021055003551 (PMC9586445; doi:10.11606/s1518-8787.2021055003551)
Supplement: Translation

# Translation: Óbitos e internações decorrentes de intoxicações por medicamentos com prescrição e isentos de prescrição, no Brasil

**Authors:** Fernanda Gross Duarte, Marcelo Neubauer de Paula, Nelzair Araújo Vianna, Maria Conceição Chagas de Almeida, Edson Duarte Moreira

**Affiliations:** I Salvador BA Brasil Pós-Graduação em Biotecnologia em Saúde e Medicina Investigativa. Instituto Gonçalo Moniz, Fundação Oswaldo Cruz. Salvador, BA, Brasil; II São Paulo SP Brasil Grupo Hypera Pharma. São Paulo, SP, Brasil; III Salvador BA Brasil Fundação Oswaldo Cruz. Instituto Gonçalo Moniz. Laboratório de Epidemiologia Molecular e Bioestatística. Salvador, BA, Brasil

**Keywords:** Medicamentos sem Prescrição, envenenamento, Medicamentos sob Prescrição, envenenamento, Hospitalização, Envenenamento, mortalidade

## Abstract

**OBJETIVO:**

Estimar a incidência de hospitalizações por intoxicação medicamentosa e a mortalidade desses agravos no Brasil, descrevendo as tendências de 2009 a 2018.

**MÉTODOS:**

Os dados de internações hospitalares e óbitos originam-se do DATASUS e os dados demográficos, do Instituto Brasileiro de Geografia e Estatística (IBGE). Foram selecionadas as internações hospitalares cuja Autorização para Internação Hospitalar (AIH) indicasse como procedimento “tratamento de intoxicação ou envenenamento por exposição a medicamento e substâncias de uso não medicamentoso”, sendo analisados apenas os casos de hospitalização por intoxicação medicamentosa. A incidência de hospitalização e a mortalidade foram calculadas separadamente para intoxicações causadas por medicamentos com prescrição (MRx) e isentos de prescrição (MIP). As taxas foram ainda estratificadas por sexo, faixa etária e região de residência no Brasil. A análise de tendência foi realizada por regressão linear generalizada pelo método de Prais-Winsten.

**RESULTADOS:**

A maioria das internações foi causada por MRx (97%), com mortalidade aproximadamente 50 vezes maior, quando comparada às internações por MIP. A tendência da incidência das internações por MRx foi estacionária, mas a mortalidade aumentou durante o período estudado, enquanto a tendência na mortalidade e na incidência de internações por MIP foi decrescente.

**CONCLUSÕES:**

As internações por intoxicação medicamentosa, sobretudo aquelas causadas por MRx, têm grande impacto e importância na saúde pública, especialmente porque esses agravos podem ser prevenidos.

## INTRODUÇÃO

Intoxicação medicamentosa é decorrente da exposição a um medicamento em doses superiores àquelas usualmente empregadas para profilaxia, diagnóstico, tratamento ou para modificação de funções fisiológicas, podendo resultar em diferentes sinais e sintomas, a depender do tipo de medicamento e do metabolismo do fármaco^1^. A intoxicação pode ocorrer de forma não intencional ou acidental, quando resulta de automedicação, de erro na dosagem, de terapêutica inadequada, de troca de medicamentos ou de ingestão involuntária; ou intencional, quando relacionada ao abuso, ao uso indevido ou à autolesão^2^. Os casos graves de intoxicação, levando a internações hospitalares e até mesmo ao óbito, constituem-se num importante problema de saúde pública^3^.

Entre 2009 e 2018, o Sistema Nacional de Informações Tóxico-Farmacológicas (Sinitox) registrou 254.135 casos de intoxicação no Brasil, com um total de 710 óbitos (0,28%). Os medicamentos foram a principal causa de intoxicação dentre todos os agentes notificados, cerca de 29% dos casos no período, seguidos por animais peçonhentos, produtos de limpeza domésticos e agrotóxicos^4^. Desde 1994, os fármacos ocupam o primeiro lugar no ranking de intoxicações e o segundo lugar em número de óbitos. Em 2016, segundo dados do Sinitox, a intoxicação acidental foi uma das principais causas das ocorrências com medicamentos, representando cerca de um terço dos casos notificados (32,7%)^4^.

Na maioria dos países desenvolvidos, a situação é semelhante à do Brasil, com medicamentos liderando as causas de intoxicações exógenas^5^. Nos Estados Unidos da América (EUA), dados nacionais de 2018 indicam que mais de dois milhões de casos de intoxicação foram causados por medicamentos^6^. Além disso, observou-se uma tendência crescente das taxas de mortalidade por intoxicações medicamentosas entre os anos de 1994 e 2010, em todas as regiões e grupos demográficos nos EUA, sendo a intoxicação acidental a principal causa (71%)^7^.

No Brasil, os medicamentos podem ser enquadrados como medicamentos de prescrição (MRx) ou medicamentos isentos de prescrição (MIP). Essa classificação foi estabelecida pela primeira vez na legislação sanitária brasileira, em 1973, quando se regulamentou o controle sanitário do comércio de medicamentos. Nessa ocasião, inseriu-se o conceito de medicamento de venda livre como aquele que independe de prescrição, estabelecendo-se diferenças quanto à rotulagem e à publicidade dos produtos prescritos^8^. Em 2016, a Agência Nacional de Vigilância Sanitária (Anvisa) definiu mais claramente os critérios para que um medicamento seja classificado como “isento de prescrição”, sendo considerados nessa categoria medicamentos de 33 grupos terapêuticos, com as devidas exceções^9^. Os critérios para a inclusão de medicamentos na lista de MIP levam em conta aspectos como: tempo de comercialização, segurança do medicamento, sintomas identificáveis, utilização por curto período de tempo, ser manejável pelo paciente, apresentar baixo potencial de risco e não apresentar dependência^9^.

O objetivo deste estudo foi estimar a incidência de hospitalizações por intoxicação medicamentosa, no geral e de acordo com o tipo de medicamento (com prescrição ou isento), bem como a mortalidade desses agravos no Brasil, descrevendo as tendências observadas nessas estimativas no período entre 2009 e 2018.

## MÉTODOS

Esse é um estudo retrospectivo para determinar as taxas de internações hospitalares e óbitos, causados por intoxicação medicamentosa no Brasil. Realizou-se uma revisão dos dados sobre intoxicações medicamentosasna base do Sistema Único de Saúde (Datasus), num período de 10 anos (data da saída em: 2009 a 2018), incluindo informações como: sexo, idade, local de residência e tipo de intoxicação medicamentosa. Os resultados referentes às intoxicações causadas por medicamentos com prescrição são apresentados separadamente daqueles causados por medicamentos sem prescrição.

Os dados de internações e óbitos são do portal do DATASUS e foram extraídos pelo *software* TabWin, fornecido pelo DATASUS. Os dados demográficos foram obtidos do Censo 2010 e das estimativas intercensitárias para os demais anos, disponibilizados pelo portal de dados do Instituto Brasileiro de Geografia e Estatística (IBGE)^10^.

Inicialmente, foram selecionadas as internações hospitalares informadas entre 2009 e 2018, que a Autorização para Internação Hospitalar (AIH) solicitava como procedimento “Tratamento de intoxicação ou envenenamento por exposição a medicamento e substâncias de uso não medicamentoso”. Posteriormente, foram mantidas apenas as internações nas quais esse procedimento era confirmado, sendo excluídas as internações consideradas para outra finalidade no relatório de alta/óbito. Os casos de intoxicação medicamentosa foram classificados, conforme os grupos terapêuticos, como causados por medicação com prescrição ou isenta, utilizando-se as informações do CID primário e secundário. Neste artigo foram analisados os casos decorrentes de intoxicação medicamentosa.

A incidência de hospitalização foi calculada dividindo-se o número de casos de internação ocorridos na população de estudo pelo número de habitantes correspondente ao período e local analisado. De maneira análoga, as taxas de mortalidade foram calculadas dividindo-se o número de óbitos pela população total em cada período do estudo. Uma vez que as taxas brutas podem ser influenciadas pela estrutura etária das populações de diferentes regiões e em períodos de tempo diferentes, as taxas estimadas foram padronizadas por faixa etária pelo método direto, utilizando-se, como padrão, a população sugerida pela Organização Mundial de Saúde (OMS 2000–2025), permitindo, assim, analisar tendências e fazer comparações desses dados^11^. A análise das frequências, taxas de hospitalizações e dos óbitos foi realizada segundo tipo de intoxicação medicamentosa classificada como: causada por medicamentos com prescrição (MRx) ou por medicamentos isentos de prescrição (MIP). As taxas foram estratificadas por sexo, faixa etária e região de residência no Brasil. A análise de tendência da série histórica foi realizada por regressão linear generalizada pelo método de Prais-Winste^12^, com correção para o efeito de autocorrelação de primeira ordem^13^. A tendência de internações hospitalares/óbitos foi considerada estacionária quando p > 0,05; declinante quando p < 0,05 e coeficiente da regressão negativo; ou ascendente quando p < 0,05 e coeficiente da regressão positivo^14^. As análises estatísticas foram realizadas utilizando-se o programa estatístico Stata (Stata Statistical Software: Release 16. College Station, TX: StataCorp LLC).

## RESULTADOS

No Brasil, ocorreram 85.811 internações hospitalares devido à intoxicação medicamentosa entre 2009 e 2018, sendo 97% delas causadas por intoxicação por MRx e apenas 3% causadas por MIP. A incidência média de internações por MRx (4,16 por 100 mil habitantes) foi muito superior à por MIP (0,13 por 100 mil habitantes), RR = 32,8 (IC95% 28,9–37,1). Houve 2.644 óbitos (3,08%) entre as hospitalizações no período do estudo. A taxa de mortalidade em casos decorrentes de intoxicação por MRx (3,11%) foi maior que a dos óbitos por intoxicação por MIP (1,93%).

A Tabela 1 mostra a frequência das internações hospitalares e dos óbitos causados por intoxicação medicamentosa segundo região e outras características sociodemográficas. A maioria das internações ocorreu no sexo feminino tanto nas intoxicações por MRx (56,7%) como naquelas por MIP (54,9%). A incidência média de hospitalizações por MRx também foi maior no sexo feminino (4,61 por 100 mil) comparado ao masculino (3,67 por 100 mil), mas essa diferença foi menor nas internações por MIP. Contrariamente, o sexo feminino representou apenas um quarto dos óbitos nas internações por MIP e pouco menos da metade daqueles nas internações por MRx. A cor da pele/raça branca foi a mais comumente reportada nas internações por MRx e MIP, em quase um terço dos casos não havia informação sobre a cor da pele. A região sudeste teve o maior número de internações por MRx, seguida da região sul e da centro-oeste. A distribuição regional das internações por MIP foi semelhante (Tabela 1).

**Tabela 1:** Frequência de hospitalizações e óbitos por tipo de intoxicação medicamentosa, Brasil, 2009 a 2018.

		Medicamentos com prescrição (MRx)				Medicamentos isentos de prescrição (MIP)				Total			
		Hospitalizações		Óbitos		Hospitalizações		Óbitos		Hospitalizações		Óbitos	
		n	(%)	n	%	n	%	n	%	n	%	n	%
Total		83.275	100	2.595	100	2.536	100	49	100	85.811	100	2.644	100
Sexo													
	Feminino	47.252	56,7	1.267	48,8	1.391	54,9	13	26,5	48.643	56,7	1.280	48,4
	Masculino	36.023	43,3	1.328	51,2	1.145	45,1	36	73,5	37.168	43,3	1.364	51,6
Faixa etária													
	< 5	7.600	9,1	40	1,5	529	20,9	4	8,2	8.129	9,5	44	1,7
	5–9	3.399	4,1	14	0,5	194	7,6	1	2,0	3.593	4,2	15	0,6
	10–14	4.623	5,6	29	1,1	144	5,7	0	0	4.767	5,6	29	1,1
	15–19	8.468	10,2	129	5,0	279	11,0	2	4,1	8.747	10,2	131	5,0
	20–29	15.679	18,8	401	15,5	476	18,8	7	14,3	16.155	18,8	408	15,4
	30–39	14.851	17,8	504	19,4	304	12,0	10	20,4	15.155	17,7	514	19,4
	40–49	11.857	14,2	543	20,9	257	10,1	4	8,2	12.114	14,1	547	20,7
	50–59	7.497	9,0	401	15,5	155	6,1	7	14,3	7.652	8,9	408	15,4
	60–69	4.331	5,2	244	9,4	88	3,5	6	12,2	4.419	5,1	250	9,5
	70 ou mais	4.970	6,0	290	11,2	110	4,3	8	16,3	5.080	5,9	298	11,3
Cor da pele/raça													
	Branca	32.814	39,4	921	36	911	35,9	11	22,4	33.725	39,3	932	35,2
	Parda	22.076	26,5	704	27	768	30,3	14	28,6	22.844	26,6	718	27,2
	Preta	2.787	3,3	93	4	77	3,0	3	6,1	2.864	3,3	96	3,6
	Outra	835	1,1	25	1	24	1,0	0	0	859	1,1	25	1,0
	Não informada	24.763	29,7	852	33	756	29,8	21	42,9	25.519	29,7	873	33,0
Regiões													
	Norte	3.868	4,6	108	4,2	142	5,6	5	10,2	4.010	4,7	113	4,3
	Nordeste	7.064	8,5	172	6,6	317	12,5	5	10,2	7.381	8,6	177	6,7
	Centro-Oeste	14.375	17,3	621	23,9	545	21,5	20	40,8	14.920	17,4	641	24,2
	Sudeste	41.522	49,9	1278	49,2	977	38,5	11	22,4	42.499	49,5	1.289	48,8
	Sul	16.446	19,7	416	16,0	555	21,9	8	16,3	17.001	19,8	424	16,0
Caráter da internação													
	Urgência	81.513	97,9	2.536	97,7	2.474	97,6	49	100	83.987	97,9	2.585	97,8
	Eletivo	1.762	2,1	59	2,3	62	2,4	0	0	1.824	2,1	59	2,2

Incidência de hospitalizações por MRx: 4,61 por 100 mil (em mulheres) e 3,67 por 100 mil (em homens).

Incidência de hospitalizações por MIP: 0,14 por 100 mil (em mulheres) e 0,12 por 100 mil (em homens).

Mortalidade por MRx: 123 por milhão (em mulheres) e 135 por milhão (em homens).

Mortalidade por MIP: 1,3 por milhão (em mulheres) e 3,7 por milhão (em homens).

A incidência de hospitalização e a mortalidade segundo faixa etária são apresentadas na Tabela 2. As intoxicações, tanto por MRx como por MIP, tiveram maior incidência de internações em menores de 5 anos, diminuindo a frequência conforme o aumento da idade, sobretudo nas hospitalizações por MIP. A média da mortalidade no período estudado foi maior nas internações por MRx do que por MIP, RR = 52,0 (IC95% 21,5–126,0). Indivíduos com 60 anos ou mais tiveram a maior taxa de mortalidade nas intoxicações por MRx e MIP, seguido pelo grupo menor de 5 anos nas internações por MIP.

**Tabela 2:** Incidência de hospitalização e mortalidade por intoxicação medicamentosa segundo faixa etária, Brasil, 2009–2018.

		Ano										Média anual	
		2009	2010	2011	2012	2013	2014	2015	2016	2017	2018		
**Hospitalização** a												
MRx (faixa etária)												
	0 a 4 anos	7,62	6,10	5,37	5,99	5,57	5,67	4,88	5,07	5,27	5,67	**5,72**
	5 a 19 anos	4,47	3,23	3,32	3,25	3,25	2,95	2,86	2,83	3,85	4,40	**3,44**
	20 a 59 anos	7,00	4,58	4,40	4,49	4,32	3,90	3,66	3,62	3,99	4,45	**4,44**
	60 anos ou mais	4,49	3,55	3,37	3,09	3,23	3,45	3,44	3,26	3,66	3,51	**3,50**
	**Total**	**6,10**	**4,22**	**4,07**	**4,11**	**4,00**	**3,73**	**3,52**	**3,48**	**3,99**	**4,37**	**4,16**
MIP (faixa etária)												
	0 a 4 anos	0,58	0,27	0,42	0,42	0,49	0,41	0,34	0,32	0,43	0,30	**0,40**
	5 a 19 anos	0,15	0,11	0,08	0,13	0,13	0,14	0,15	0,13	0,13	0,14	**0,13**
	20 a 59 anos	0,20	0,11	0,12	0,10	0,11	0,09	0,09	0,08	0,08	0,08	**0,11**
	60 anos ou mais	0,14	0,08	0,10	0,10	0,05	0,08	0,08	0,06	0,04	0,04	**0,08**
	**Total**	**0,21**	**0,12**	**0,13**	**0,13**	**0,13**	**0,12**	**0,12**	**0,11**	**0,10**	**0,10**	**0,13**
**Mortalidade** b												
MRx (faixa etária)												
	0 a 4 anos	66	37	22	15	7	23	15	23	38	53	**30**
	5 a 19 anos	38	26	30	33	35	31	34	32	57	44	**36**
	20 a 59 anos	221	137	155	155	148	152	155	160	164	191	**164**
	60 anos ou mais	214	151	134	181	207	215	215	173	239	247	**198**
	**Total**	**162**	**103**	**112**	**119**	**119**	**124**	**126**	**125**	**143**	**159**	**129**
MIP (faixa etária)												
	0 a 4 anos	7,4	14,8	0	0	0	7,7	0	0	0	0	**3,0**
	5 a 19 anos	0	0	0	0	6,2	0	0	0	0	0	**0,6**
	20 a 59 anos	5,6	3,7	5,4	1,8	0,9	2,7	0,9	0	3,4	0,8	**2,5**
	60 anos ou mais	4,5	8,9	13,0	3,9	0	7,3	0	10,2	3,3	3,1	**5,4**
	**Total**	**4,1**	**4,1**	**4,6**	**1,5**	**2,0**	**3,0**	**0,5**	**1,5**	**2,4**	**1,0**	**2,5**

a Incidência de hospitalização por 100 mil habitantes.

b Mortalidade por 100 milhões de habitantes. Medicamentos isentos de prescrição (MIP); Medicamentos com prescrição (MRx).

A Tabela 3 apresenta a incidência de hospitalização e a mortalidade, padronizadas para a distribuição da população mundial (OMS 2000–2025), segundo as regiões do país. No período do estudo, a maior incidência média de internações por MRx ocorreu na região sul e sudeste, enquanto as regiões norte e nordeste apresentaram as menores taxas. Nas hospitalizações por MIP, a incidência média foi maior na região centro-oeste e sul. De forma semelhante, as regiões norte e nordeste apresentaram as menores taxas. A mortalidade observada nas intoxicações por MRx foi mais elevada nas regiões sudeste e sul, enquanto as causadas por MIP teve maior taxa na região nordeste.

**Tabela 3:** Incidência de hospitalização e mortalidade por intoxicação medicamentosa segundo região, Brasil, 2009–2018.

		Ano										Média anual	
		2009	2010	2011	2012	2013	2014	2015	2016	2017	2018		
**Hospitalização** a												
MRx (por região)												
	Norte	3,23	2,54	2,80	3,72	3,71	1,62	1,29	1,17	1,52	1,33	**2,29**
	Centro-oeste	9,28	5,91	5,34	5,17	4,81	3,84	2,99	3,49	3,94	3,63	**4,84**
	Nordeste	4,83	2,83	2,73	2,79	2,42	2,34	2,18	2,00	2,09	2,43	**2,66**
	Sudeste	6,64	5,09	5,08	4,88	4,85	4,94	4,38	4,61	5,18	5,76	**5,14**
	Sul	8,35	5,53	4,81	4,94	5,35	5,38	4,96	5,81	7,18	7,72	**6,00**
	Brasil	**6,10**	**4,23**	**4,08**	**4,10**	**3,99**	**3,72**	**3,49**	**3,47**	**3,98**	**4,36**	**4,15**
MIP (por região)												
	Norte	0,13	0,05	0,06	0,10	0,11	0,12	0,09	0,02	0,08	0,04	**0,08**
	Centro-oeste	0,57	0,26	0,21	0,20	0,19	0,24	0,10	0,11	0,13	0,15	**0,22**
	Nordeste	0,17	0,11	0,15	0,10	0,11	0,08	0,09	0,07	0,05	0,06	**0,10**
	Sudeste	0,17	0,09	0,10	0,12	0,12	0,12	0,12	0,12	0,10	0,09	**0,12**
	Sul	0,27	0,17	0,15	0,21	0,17	0,18	0,21	0,19	0,20	0,21	**0,20**
	Brasil	**0,21**	**0,12**	**0,13**	**0,13**	**0,14**	**0,13**	**0,12**	**0,11**	**0,11**	**0,11**	**0,13**
**Mortalidade** b												
MRx (por região)												
	Norte	68	80	58	74	67	49	53	48	128	53	**68**
	Centro-oeste	222	130	111	113	177	103	116	71	148	111	**130**
	Nordeste	224	76	105	131	120	103	91	135	101	147	**123**
	Sudeste	153	137	149	154	155	191	148	176	194	226	**168**
	Sul	205	136	123	124	132	157	134	139	236	249	**164**
	Brasil	**158**	**101**	**108**	**115**	**115**	**120**	**121**	**120**	**139**	**155**	**125**
MIP (por região)												
	Norte	0	5,73	0	0	10,94	5,41	0	0	0	5,52	2,76
	Centro-oeste	7,07	0	25,42	0	0	6,60	0	0	0	0	3,91
	Nordeste	5,60	13,52	6,79	1,83	1,54	6,11	0	3,02	3,56	2,99	4,50
	Sudeste	5,70	1,22	2,05	2,03	0	1,18	1,08	1,96	1,15	0	1,64
	Sul	0	0	7,14	3,54	2,98	3,48	0	5,78	9,14	0	3,21
	Brasil	**4,09**	**4,24**	**4,43**	**1,46**	**2,06**	**3,02**	**0,46**	**1,49**	**2,32**	**0,94**	**2,45**

a Incidência de hospitalização por 100 mil habitantes padronizada para a distribuição da população mundial (OMS, 2000–2025).

b Mortalidade por 100 milhões de habitantes padronizada para a distribuição da população mundial (OMS, 2000–2025).

Medicamentos isentos de prescrição (MIP); Medicamentos com prescrição (MRx).

O número total de hospitalizações e óbitos, os coeficientes de regressão e a tendência de hospitalizações e de mortalidade por tipo de intoxicação medicamentosa, segundo região brasileira, são apresentados na Tabela 4. No período de 2009 a 2018, a tendência da incidência de hospitalizações por intoxicação medicamentosa por MRx foi estacionária, variando de ascendente na região sul a decrescente nas regiões centro-oeste e nordeste. Enquanto a incidência de internações por MIP teve tendência decrescente no Brasil, especialmente nas regiões centro-oeste e nordeste. A tendência da mortalidade das intoxicações por MRx foi ascendente no Brasil, inclusive nas regiões sul e sudeste. Já a mortalidade das intoxicações por MIP mostrou tendência decrescente no país.

**Tabela 4:** Hospitalizações e óbitos, coeficiente de regressão e tendência de hospitalizações e mortalidade por intoxicação medicamentosa segundo região, Brasil, 2009–2018.

		n	Coeficiente	IC 95%	p	Tendência
**Hospitalização**						
MRx (por região)						
	Norte	3.868	−0,268	−0,586 a 0,050	0,087	Estacionária
	Centro-oeste	7.064	−0,224	−0,407 a −0,041	0,023	**Decrescente**
	Nordeste	14.375	−0,080	−0,146 a −0,014	0,024	**Decrescente**
	Sudeste	41.522	0,124	−0,020 a 0,268	0,082	Estacionária
	Sul	16.446	0,514	0,328 a 0,700	0,000	**Ascendente**
	Brasil	83.275	0,049	−0,072 a 0,169	0,371	Estacionária
MIP (por região)						
	Norte	142	−0,002	−0,0153 a 0,010	0,605	Estacionária
	Centro-oeste	317	−0,014	−0,026 a −0,002	0,025	**Decrescente**
	Nordeste	545	−0,010	−0,011 a −0,009	0.000	**Decrescente**
	Sudeste	977	−0,000	−0,003 a 0,002	0,705	Estacionária
	Sul	555	0,004	−0,000 a 0,009	0,052	Estacionária
	Brasil	2.536	−0,004	−0,005 a −0,002	0,001	**Decrescente**
**Óbitos**						
MRx (por região)						
	Norte	108	0,283	−5,478 a 6,045	0,911	Estacionária
	Centro-oeste	172	−3,446	−9,001 a 2,109	0,186	Estacionária
	Nordeste	621	1,772	−1,879 a 5,423	0,289	Estacionária
	Sudeste	1.278	6,680	3,959 a 9,401	0,001	**Ascendente**
	Sul	416	15,527	6,757 a 24,297	0,004	**Ascendente**
	Brasil	2.595	5,885	3,635 a 8,136	0,000	**Ascendente**
MIP (por região)						
	Norte	108	0,283	−5,478 a 6,045	0,911	Estacionária
	Centro-oeste	172	−3,446	−9,001 a 2,109	0,186	Estacionária
	Nordeste	621	1,772	−1,879 a 5,423	0,289	Estacionária
	Sudeste	1.278	6,680	3,959 a 9,401	0,001	**Ascendente**
	Sul	416	15,527	6,757 a 24,297	0,004	**Ascendente**
	Brasil	2.595	5,885	3,635 a 8,136	0,000	**Ascendente**

Medicamentos isentos de prescrição (MIP); Medicamentos com prescrição (MRx).

## DISCUSSÃO

No decênio avaliado neste estudo (2009–2018), a maioria das internações por intoxicação medicamentosa foi causada por MRx (97%), uma incidência 32 vezes maior que a de hospitalizações por MIP. Apesar dessa grande diferença, indicando o papel preponderante das MRx comparadas às MIP nos casos graves, os dados de comercialização durante a segunda metade da década estudada (2014–2018) mostram que foram vendidas menos doses de MRx (7,2 bilhões) do que de MIP (7,7 bilhões)^15^. A isenção de prescrição baseia-se sobretudo no baixo potencial de toxicidade desses produtos, que têm reações adversas reversíveis após suspensão de uso e reações graves apenas com a administração de grande quantidade do produto, além de apresentarem janela terapêutica segura^16^. Possivelmente, este perfil de segurança é responsável pelo menor número de intoxicações graves e hospitalizações causadas por MIP. A mortalidade muito maior observada nas internações por MRx, cerca de 50 vezes maior, sustenta essa argumentação.

A ANVISA é responsável pela regulamentação de propaganda, publicidade, informação e outras práticas com objetivo de divulgação ou promoção comercial de medicamentos^17^, incluindo também os materiais elaborados pela indústria farmacêutica com a finalidade de apresentar aos profissionais prescritores e dispensadores. Os resultados desta pesquisa, indicando mortalidade muito mais elevada no uso de medicamentos com prescrição, têm implicações importantes, que justificam o aprimoramento dessa regulamentação para prevenir a automedicação e o uso de medicamentos de forma não racional.

As internações foram mais comuns no sexo feminino, enquanto a mortalidade foi maior no masculino, tanto nas intoxicações por MIP como por MRx. Mota et al. (2012), analisando o decênio de 1996 a 2005, também observaram maior prevalência de óbitos por intoxicações medicamentosas em homens^18^. As internações por intoxicação medicamentosa foram mais frequentes em menores de 5 anos de idade, independentemente do tipo de medicação. A ingestão acidental de medicamentos é mais comum nessa faixa etária^19,20^, além disso, o menor peso corporal nesse grupo torna-os mais vulneráveis à intoxicação com doses relativamente menores^21,22^. Quanto às taxas de mortalidade, elas foram maiores nos mais idosos, semelhante ao observado em outros estudos^23,24^. É possível que o uso mais frequente de medicamentos e a susceptibilidade aumentada à toxicidade, associados à diminuição da capacidade de metabolização e excreção de medicamentos, decorrente do avanço da idade, possam ter contribuído para maior mortalidade observada em idosos^24,25^.

A incidência das internações por MIP e MRx variou no Brasil, onde as regiões sul, centro-oeste e sudeste apresentaram as maiores taxas e as regiões norte e nordeste as menores. A mortalidade das intoxicações variou segundo a região do país, no sudeste e sul ocorreram as maiores taxas de mortalidade por MRx, enquanto as regiões nordeste e centro-oeste apresentaram a maior mortalidade por MIP. Existem evidências no Brasil da associação entre maior poder aquisitivo e maior utilização de medicamentos^26^, essa pode ser uma explicação para taxas mais elevadas e crescentes de intoxicação medicamentosa nas regiões sul e sudeste onde a renda per capita da população é maior do que as demais regiões do país^27^. Desse modo, as diferenças observadas podem ser desdobramentos das desigualdades na disponibilidade e no acesso a medicamentos das populações residentes nessas regiões. Podem ser também consequências de diferenças nas coberturas dos serviços de saúde responsáveis pela notificação dos casos e dos óbitos por intoxicações medicamentosas nas diferentes regiões no Brasil.

A tendência da incidência das internações por MRx foi estacionária, mas a mortalidade aumentou durante o decênio, enquanto houve tendência decrescente na mortalidade e na incidência de internações por MIP no mesmo período. O perfil epidemiológico da população brasileira vem se modificando com aumento da prevalência de doenças crônicas e consequente uso de medicamentos com maior frequência^28,29^. A polifarmácia tem se tornado um problema entre idosos, no Brasil, estima-se que 82% das pessoas acima de 60 anos façam uso de ao menos um medicamento^30^. Embora esses medicamentos possam aumentar a qualidade e a expectativa de vida, eles também podem trazer riscos a essa população devido às interações medicamentosas e à alteração da fisiologia, causada pela senescência^31^. Isto pode ser um dos fatores responsáveis pela manutenção da incidência das internações e pelo aumento da mortalidade nos casos de intoxicação por MRx.

### Limitações e Méritos

A natureza retrospectiva desse estudo baseado em banco de dados preexistente, em que as informações disponíveis são limitadas, impediu que algumas características relativas às circunstâncias, às causas e ao tratamento das internações por intoxicação medicamentosa pudessem ser investigadas. Além disso, as informações referem-se apenas aos serviços de saúde públicos, não abrangendo os serviços privados, portanto, as taxas produzidas estão subestimadas. No entanto, a cobertura nacional dos dados, incluindo um período de vários anos, permitiu comparações entre diferentes regiões e forneceu medidas de tendência para a incidência das internações por intoxicação medicamentosa, assim como para a mortalidade.

## CONCLUSÕES

As internações por intoxicação medicamentosa têm grande impacto e importância na saúde pública, merecendo atenção especialmente por sua natureza potencialmente prevenível. Apesar do maior acesso a medicamentos com isenção de prescrição, a quase totalidade das hospitalizações e dos óbitos no Brasil entre 2009 e 2018 ocorreu em decorrência de intoxicações causadas por medicamentos com prescrição obrigatória. Nossos resultados reforçam a necessidade de estudos analíticos para identificar os determinantes e prevenir a ocorrência das intoxicações medicamentosas.
